# Research on the mechanism of short video information interaction behavior of college students with psychological disorders based on grounded theory

**DOI:** 10.1186/s12889-023-17211-4

**Published:** 2023-11-16

**Authors:** Wang Linlin, Huang Wanyu, Li Yuting, Qiao Huimin, Li Zhi, Jiang Qinchen, Wang Tingting, Wang Fan, Pan Minghao, Zhu Wei

**Affiliations:** 1 Medical College, Xinyang Normal University, 237 Nanhu Road, Xinyang City, 464000 Henan Province China; 2 School of Public Health Wuhan University, Wuhan University, 115 Donghu Road, Wuhan City, 430071 Hubei Province China; 3 Hangzhou Wickham International School, Hangzhou City, 311000 Zhejiang Province China; 4 Yantai Automobile Engineering Professional College, Yantai City, 265500 Shandong Province China; 5 Xinyang Central Hospital, Xinyang City, 464000 Henan Province China; 6 Zhoukou Normal University, Zhoukou City, 466000 Henan Province China; 7 School of Information Management, Wuhan University, Wuhan City, 430072 Hubei Province China

**Keywords:** College students with psychological disorders, Short video, Information interaction, Grounded theory

## Abstract

**Background:**

The utilization of short videos by individuals often leads to the emergence of information exchange behavior. Previous studies have shown that certain students with psychological disorders exhibit addictive tendencies towards short video-related software. Therefore, it is essential to address the psychology and behavior of college students with psychological disorders while engaging with short videos. This study aims to explore the mechanism of short video information interaction behavior among college students with psychological disorders.

**Methods:**

We conducted semi-structured interviews with 30 college students afflicted by psychological disorders in a prefecture-level city in Henan Province, China from September to December 2022. Based on the Grounded theory, we encoded 30 text materials across three levels to explore the mechanism of short video information interaction behavior among college students with psychological disorders, and subsequently build a model framework.

**Results:**

The findings of this study suggest that college students with psychological disorders exhibit negative cognition tendencies that can lead to strongly negative emotions, excacerbated by a lack of social support. These adverse factors collectively drive the consumption of short video content in this demographic, providing a virtual environment where they can fulfill their unmet social needs. Therefore, the mechanism governing short video messages interaction among college students with psychological disorders encompasses negative cognitive tendencies, negative emotions, lack of social support, post-video-watching behaviors, and the gratification of social needs within the confines of a virtual environment.

**Conclusions:**

This study comprehensively analyzes the motivation and complexity of college students with psychological disorders in short video interaction. Although short videos provide this group with some ways of self-expression and emotional support, they still have a negative impact on their physical and mental health. The short video interaction of college students with psychological disorders is affected by many factors, including their negative cognitive tendencies, negative emotions, lack of social support, post-video-watching behaviors, and the gratification of social needs within the confines of a virtual environment. These findings deepened our understanding to the mechanism of short video information interaction behavior among college students with psychological disorders, also provided us with guidance on facilitating the proper use of short video and maintaining the mental health. In future researches, researchers can discuss more about intervention measures to help this demographic cope with the challenges from short video interaction.

**Supplementary Information:**

The online version contains supplementary material available at 10.1186/s12889-023-17211-4.

## Introduction

The short video is a new type of internet transmission method, with a duration generally lasting less than 5 min. It integrates text, sound, and pictures, and caters to the diverse entertainment needs of the public [1]. The number of short video users in China has exceeded 10.26 billion, accounting for 95.2% of the total number of Internet users [2]. On short video platforms, users can establish connection with real-life contacts or other unfamiliar users through short videos and engage in various activities such as liking, commenting, sharing, messaging, and bookmarking after browsing videos posted by others [3].

Information exchange refers to the exchange of information between participating entities through a set of information interfaces (channels) [4]. In existing research, research on online user information interaction behavior mainly focuses on interaction behavior patterns [5], network characteristics of interaction behavior [6], and interaction influencing factors [7–9]. Savolainen [10] believes that information exchange behavior in Q&A communities or online forums is a dialogue interaction behavior between network information resource providers and information seekers. Barabasi [11] believes that once information behavior occurs between users, information exchange occurs and the structure of the network is formed. Saike et al. [12] believes that if there are more similarities between people, the more likely information exchange will happen. Chung et al. [13] suggest that text sentiment can affect information interaction between users. Information interaction is a complex process for users to obtain and utilize information [14] and involves three aspects: users, information objects, and systems. Interaction is essentially the circulation of information, and the implementation of this kind of circulation requires the creation, absorption and feedback provided by users [15]. Users can perceive the presence of others and themselves when they interact with others in a virtual space [16]. It allows them to experience emotional satisfaction and psychological recognition. The concise transmission of information and the efficiency of “viral-like” dissemination have made short videos a new way of information exchange [17, 18].

College students have become the main force in watching short videos [19] and they are engaging in sustained information exchange behavior [20]. Meanwhile, over the past few decades, there has been a significant rise in the prevalence of mental disorders, including depression and anxiety, among university students [21–24]. In China, the overall prevalence of depression among university students is 23.8% [25]. Depressive emotions may lead to a sense of hopelessness among these students [26] and unwillingness to seek external help [27], which may result in delayed treatment or even suicidal behavior [28]. University students often encounter psychological issues such as anxiety, depression, and interpersonal sensitivity. Therefore, college students in psychological distress deserve our attention. Research indicates that the utilization of “short video + social media” platforms such as Snapchat, Facebook, or TikTok may may elevate their risk of developing depression [29]. Furthermore, Some college students with psychological disorders are addicted to short video-related software, which has a negative impact on their physical and mental health [30]. The college stage can be a period of high incidence of psychological disorders. Since short video has become an indispensible part of their lives, the relevant psychological and behavioral mechanisms of college students demand our focused attention. This study will offer valuable insights for future research and practical applications, while also contributing to the enhancement of the mental health of college students struggling with psychological challenges and mitigating any potential negative impact of short videos.

## Methods

### Study approach

Charmaz’s grounded theory of constructivism is adopted in this study [31, 32], which belongs to the category of qualitative research and aims to study social interaction or experience, developing a theory on the basis of data [33]. The research attempts to elucidate the impact mechanism of the short video information interaction among the college students with mental disorders. Therefore, Grounded theory research methods are used to systematically extract factors that affect the short video information interaction among college students with mental disorders.

#### Ethical considerations

Approval for this study was obtained through the Ethics Committee of Xinyang Normal University (Study ID: XFEC-2022-019).

#### Recruitment

From September to December 2022, undergraduate students from 22 universities were selected as interviewees. Based on the results of National Psychological Survey of College Students, we selected 30 students in need of focus for further interviews. Inclusion criteria: (1) full-time undergraduate students, (2) experienced in watching short videos, (3) voluntarily participated in this study, (4) a group of college students who tested positive for anxiety, depression, or suicidal idea. Exclusion criteria: (1) Individuals with communication disorders, (2) Individuals who have received psychological counseling or intervention, (3) Individuals who are currently taking psychotropic medications. In the early stages of the study, purposive sampling was adopted based on the research purpose, and it was considered reached theoretical saturation when there were no new factors affecting the cognition and judgment of the interviewees in the short video information interaction [34].

#### Data collection

This article adopts a one-to-one semi-structured interview method to collect data, field notes and observation methods are used at the same time. The main content of the interview includes: (1) How is your recent emotional state? (2) What is your motivation for participating in a short video information exchange? (3) What factors will affect your short video information interaction? (4) What are your strongest thoughts and feelings during the short video messages interaction? (5) What impact do short video consumption have on your psychology, life, and learning? Finally, a comprehensively analysis of all data is conducted to ensure the accuracy of data analysis. According to the final results, the interview time for each interviewee in this study ranged from 16 to 35 min, with an average of 24 min, and a total of more than 41,000 words were transcribed into text manuscript.

#### Data analysis

The research method of Constructivism Grounded theory is adopted to analyze the data [35]. Open coding is the initial phase of the Grounded theory process, which yield 281 original sentences and 41 original concepts in total. In the axial encoding process, 12 main categories are formed. Selective encoding is a kind of axial encoding, it continues at a higher level of abstraction, and resulting in five main categories: negative cognitive tendencies, negative emotions, lack of social support, post-video-watching behaviors, and the gratification of social needs within the confines of a virtual environment. Based on these factors, researchers will further construct a behavioral model of the short video information interaction among college students with psychological disorders. The specific coding process is shown in Appendix 1.

## Results

### Participant characteristics

Participants in this research included 30 college students with psychological disorders, with 23 girls and 7 boys. The average age of the participants is 19 years old. Shown in Table 1.

**Table 1:** General Information of Interviewees(n = 30)

Number	Gender	Age	Place origin	Year of smartphone usage(years)	Does watching short videos have an impact on life and learning	Short video viewing duration/day
N1	female	21	countryside	7	True	2–3
N2	female	19	city	8	True	2
N3	female	21	countryside	7	True	2–3
N4	female	20	countryside	4	True	3–4
N5	female	19	city	6–7	True	1
N6	female	20	city	6–7	False	8–9
N7	female	18	city	7	True	3–4
N8	female	20	city	7	True	5–6
N9	male	18	countryside	4	True	1
N10	female	20	countryside	5	True	1
N11	female	19	countryside	7	True	3–4
N12	female	19	countryside	5	True	3
N13	female	20	city	5	True	2
N14	female	21	city	5	True	2–3
N15	female	21	countryside	7	True	< 1
N16	male	19	countryside	5	True	3
N17	female	18	countryside	3	True	4
N18	female	19	countryside	4	True	5
N19	male	18	city	6	True	6
N20	female	20	countryside	5	True	6
N21	male	21	countryside	4	True	4
N22	female	21	countryside	4	True	6
N23	female	20	countryside	3	True	4
N24	male	19	countryside	2	True	5
N25	female	20	city	4	True	4
N26	female	19	countryside	3	True	4
N27	male	18	city	5	True	5
N28	female	20	countryside	6	True	6
N29	female	21	countryside	3	True	4
N30	male	19	countryside	4	True	6

#### Construction of short video information interactive behavior mechanism model for college students with psychological disorders

**Fig. 1:**
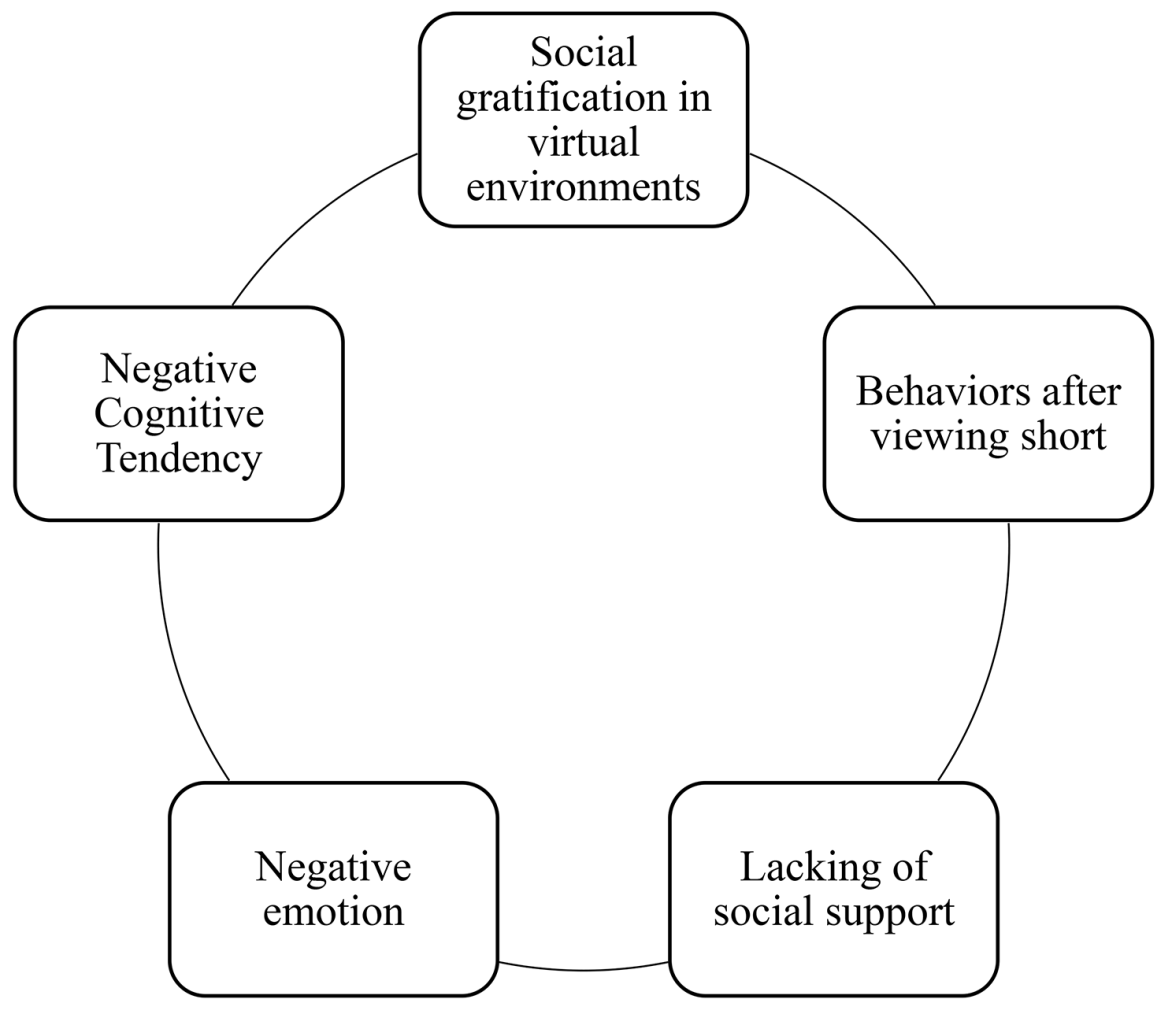
Interactive behavior model of short video information of college students with psychological disorders

The findings of this study show that college students with psychological disorders exhibit negative cognitive tendencies that can lead to strongly negative emotions [36, 37], excacerbated by a lack of social support [38, 39]. Therefore, they are trying to fulfill their unmet social needs in the virtual environment provided by short video [40]. After analyzing the results of this study, this paper proposes that the short video information interaction behavioral mechanism of college students with psychological disorders includes five factors: negative cognitive tendency, negative emotion, social support, post-video-watching behaviors, and the gratification of social needs within the confines of a virtual environment. See Fig. 1.

### Negative cognitive tendency

#### Self-competence denial

In this study, the self-competence denial of college students with psychological disorders serves as the direct manifestation of their negative cognitive tendencies, which is particularly evident in the concerns, depression, anxiety, and other symptoms expressed by the participants during interviews. Students with psychological disorders are apt to compare the lives shown by others in short videos with their own, resulting in a large psychological gap and self-doubts. The self-competence denial of college students with psychological disorders has the following characteristics: they said they were striving for perfection but also were afraid of failure. They had a desire for success but they were not confident of their own ability. When encountered with a new task (such as an exam), they would be excessively worried, self-doubting and anxious. They would repeatedly denied their ability rather than actively solved the problems. Thus watching short video became a way of releasing pressure, resolving sadness, and seeking sympathy. College students with psychological disorders indicated that if the content of short videos could arouse their own emotional resonance, they were more willing to participate in information interaction.

*Because my academic performance is not good, I want to work hard when I am watching inspirational videos. But I’m weak in self-control. I can only keep my enthusiasm for a short while, then I can not raise my interest in studying anymore. Watching short videos can make me feel relaxed.(N18)Compared with other students, I feel that I am not beautiful enough, I don’t have good body shape, and my academic performance is so poor. Watching short videos can help me temporarily forget unpleasant things. (N6) In my daily life, I will watch some funny short videos, and then laugh, that will make me feel better. Sometimes, I will watch some short videos of chicken soup for the soul (to relax and release psychological pressure). (N1)*

#### Negative life events

Negative life events refers to a kind of stressful life events that will cause individuals’ negative emotional experience such as anxiety, depression and low self-esteem, including aspects of learning, life, physical health, interpersonal relationship. The negative life events of college students with psychological disorders have the following characteristics: They indicated that the negative life events mainly came from excessive academic pressure and interpersonal relationship tensions [41], such as the friction or contradiction with classmates due to trivial events. They lacked of effective ways and were unable to solve the problems in time. Therefore, the continued frustration made it difficult for them to get rid of these negative emotions. They said they were not willing to reveal their true inner thoughts to their families or classmates, but were more inclined to use short video interaction to address their emotional troubles. That facilitated more information interaction.

*For example, when your roommate takes it out on you for no reason and you get really angry. Sometimes they are very noisy in the dormitory. Sometimes they snore at night, you can’t sleep and feel uncomfortable. Then you don’t know what to do, so you can only watch some short videos and try to please yourself. My parents are not well educated and they are busy with their work. Every time, I can only talk to them for a while before we hang up. I don’t have a close relationship with my classmates, so I dare not tell them something for fear that they will tell others, and then more people will know. However, in short videos, others don’t know me, and I won’t divulge my own privacy. I can express my own ideas and tell others about my unhappiness. (N11)*

#### Negative academic attitude

College students with psychological disorders are in adolescence. Lacking of certain life and social experience makes it easy for them to be stimulated and influenced by various external environmental factors. Due to the heavy study pressure, insufficient learning methods, disturbing interpersonal relations and so on, they may feel tired of studying and living, which can ultimately result in a negative academic attitude. College students with psychological disorders have expressed that the original intention of watching short videos was to relax and decompress, and to meet their emotional needs [42]. Short videos will attract users’ attention within a few seconds and keep them engaged and watching [43], which is further promoting the interaction of information.

*I feel like I just can not stop watching short videos, time passes quickly when I am indulge in short videos, it feels like I am in a whirlpool of time. (N3) Watching some relaxed, humorous, and funny short videos somewhat more decompression, which can make me feel happy temporarily, and my mind can be temporarily empty. (N3) I have a sense of powerlessness because I am not professional enough in my major subject, and I may have some grammar problems that I really don’t understand. Watching short videos takes my mind off things for a while, but sometimes I lose track of time. (N6)*

#### Negative emotion

College students with sychological disorders are prone to negative emotions due to external factors such as interpersonal relationship issues, learning pressure, adverse cognition and so on [44]. College students are not good at communication and dare not to express themselves to others. They often adopt a negative attitude towards the daily events, which can make psychological disorders more severe. The stress caused by academic performance can aggravate the teenager*’*s negative emotions, leading to their excessive addiction to short videos [45].

*I feel confused about my future and I do not know what to do. Watching short videos makes me stop thinking about it, just muddling along without thinking about tomorrow. (N3) Watching interesting videos helps me forget about my worries and time, and sometimes I feel much better after laughing.(N25)*.

#### Lacking of social support

The absence of social support increases the risk of depression in adolescents [46]. Research indicates that adolescents suffering from depression are more inclined to seek social support on social networks, often experienceing enhanced levels of support within these online platforms [38].College students with psychological disorder are more likely to feel lonely when they lack effective social support in real life. College students can gain a sense of belonging and identitythrough short videos [47]. College students said that they were eager to express their real emotions, but at the same time, they were also worried that expressing their real emotions would lead to adverse consequences [48]. Therefore, they tended to choose a short video platform to interact with strangers to alleviate their inner conflicts. In addition, liking and commenting on short videos were also a special social behavior. College students said that participating in the interaction could make them feel a sense of achievement, satisfaction, identification, and some emotional support [49].

*I will feel anxious and sad when I face the problems that I can’t solve. I feel embarrassed to tell my classmates and family members about these private things. I am also afraid that my privacy will be leaked. When I see short videos that are similar to my life, I will send a private message to the blogger, asking him or her for advice. (N9) Sometimes I will express my opinion in the comments, and when I see a lot of people give me a thumbs up, I will feel a sense of accomplished.(N15)*.

### Behaviors after watching short videos

#### Content absorption behavior

College students expressed that through the short video interaction behaviour, they could not only obtain new knowledge and learning methods but also possess the ability to extract implicit knowledge that was difficult to be fully obtained in real life. A good interactive experience could meet the information interaction outcomes expectations of psychologically impaired college students, so as to accumulate positive emotions and promote continuous information interaction behavior among college students with psychological disorders. In the meantime, college students with psychological disorders said that if the author or organization were professionally competent enough to operate an official account, they would be more willing to participate in information interaction.

*Sometimes I will also browse some short videos about learning, and then I can learn some knowledge. Most of the knowledge can be gained from short videos.(N7). Watching short videos can help me improve some of my life skills. I can also follow the way of taking videos in my daily life.(N29) I like to follow some bloggers who share the videos about outfit, fitness, or videos, etc. I will see if there are any updates, and looking forward to better videos.(N11)*.

#### Impulsive and extreme behavior

College students said that once they recognized that they were addicted to watching short videos, they would uninstall the short video software, but suddenly stopping watching short videos could lead to more negative emotions, such as anxiety and depression. After stopping watching short videos, the fear of missing out would arise. Therefore, they continued to use short videos. Excessive dependence on the Internet leads to the emergence of addiction symptoms. This negatively affects the psychological and emotional development of adolescents [50]. When college students are indulged in watching short videos, they become addicted to short videos, which has a negative impact on their learning motivation [30]. In addition, college students with psychological disorders follow various bloggers that they like. The bloggers’ timely updates and feedback can bring a good interactive experience to their users, meet users’ expectations for information interaction, and promote users’ information interaction behavior.

*I can’t control myself, but I will blame myself and feel like a failure.(N5) I can not stop watching short videos, that makes me unable tp focus on my studies. Once I uninstall it, I will feel bored and anxious. Then I will download it again and again.(N19)*.

### Social gratification in virtual environments

#### Self-expression in a virtual environment

Due to their sensitivity and suspicious college students attach too much importance to others’ opinions and evaluations. As a result, they struggle to express their inner thoughts and often exhibit extreme reactions to others’ words and deeds during interpersonal communication, which often impairs effective communication. Likes, comments, bullet comments, and other functions of the short video platform create a virtual social mode. Users can voice their opinions on the content of the short video at anytime without affecting the communication of users in real life [51]. This satisfies the need for self-expression of college students with psychological disorders and stimulates their desire for expression. In interviews, college students stated that poor interpersonal communication would increase their sense of loneliness and depression. In short videos, they could establish virtual interpersonal relationships with other unfamiliar users and freely expressed their opinions, continuously enhancing their willingness to exchange information.

*Sometimes I get thumbs up from netizens, I am so happy that I always go and check if there is anyone who gives me a thumbs up or replies. (N14) I am introverted and I am not willing to communicate with others. The relationship between new classmates and roommates will make me feel nervous. On the short video, I do not need to communicate with others face to face, and I am not afraid of saying anything wrong. Because other people do not know who I am, so I can express my ideas. (N26)*

#### Emotional resonance in virtual environments

In the interviews, university students with psychological disorders expressed that they sought emotional resonance by sharing personal experiences with others who had similar backgrounds through short videos, in order to gain respect and recognition. In daily life, these students often adopt a negative attitude towards things, exaggerating the negative effects on them, which can lead to negative emotions. In order to vent and satisfy their emotional needs, they interacted with other users through short videos to gain understanding and support. Additionally, certain short video content was compatible with cognitive evaluations, and students with psychological disorders could easily obtain emotional resonance and a special emotional experience, ultimately promoting continuous information exchange behavior.

*Feeling unproductive, feeling like I am repeating the same day every day, feeling a bit wasted, feeling a bit decadent, feeling that I can’t do anything. There are many people on short video platforms who have similar experience to me, I find they understand me.(N2) Sometimes when I come across a video that is similar to my current situation, I can empathize with these people so I will click to see the blogger’s experiences, and I will also send private messages or leave comments to encourage them. (N13)*

#### Emotional acquisition in virtual environments

University students with psychological disorders, in general, tend to exhibit a reluctance towards engaging in real-life social activities. This is due to the fact that they often ebdure emotions such as inferiority and depression, which in turn prompts them to avoid socializing. When facing negative events, they tend to adopt a negative attribution style, that is, attributing the cause of negative events to themselves or others, and they are unable to extricate themselves from negative emotions and events. Faced with negative events, university students with psychological disorders will make more negative evaluations about themselves, and lead to more depression. Once Internet users discover that online activities can bring them psychological rewards, their expectations of receiving such rewards will drive them to continue using the Internet [52, 53]. To some extent, humorous short videos can alleviate feelings of loneliness and relieve stress, catering to the emotional needs of university students with psychological disorders. These students claimed that engaging in interactive activities helped release their stress and dissolve negative emotions.

*The exam results are not satisfying, and I don’t have a proper learning method, sometimes my teacher is speaking too fast and I cannot digest the information in time, I will feel that I am a failure. Watching short videos can distract me from these things. (N15) Because I started from zero in this major, and there may be some grammatical problems that I really don’t understand, I feel powerless. Watching short videos can make me relax and relieve my anxiety. (N6) I’m always attracted to short videos. When I’m watching them, I feel happy. Watching some interesting short videos can make me feel better. (N3)*

#### Reality avoidance in the virtual environment

Avoidance strategy refers to the individual’s way of coping with anxiety by excluding situations that cause anxiety. This may involve denial, avoidance, daydreaming, etc., which can directly or indirectly impact information exchange behavior. Previous research has shown that users can temporarily alleviate real-life stress by watching short videos [54]. Compared to the general population, college students with psychological disorders are more inclined to employ avoidance strategies when confronted with negative life events. They tend to immerse themselves in short videos as a temporarily escape from their problems, thereby facilitating sustained engagement in information exchange behavior.

*Sometimes when I don’t get along well with my roommates and can’t understand what the teacher is saying in the class, I will feel uncomfortable. Watching short videos can make me feel better and forget about those bad things. (N13)*

## Discussion

### Major findings

Based on Grounded Theory, in-depth interviews were conducted to analyze the mechanism of psychological disorders in college students’ short video information interaction behavior. The results showed that the short video information interaction behavior of these students with psychological disorders includes negative cognitive tendency, negative emotions, insufficient social support, post-viewing behavior of short videos, and virtual social satisfaction. In addition, there are mutual influences and circular effects between these fators. These findings not only expand the research on short video information interaction behaviors among college students with psychological disorders, but also contribute to better understand the relationship between college students with psychologocal disorders and short video interaction.

There is a strong correlation between the negative cognitive tendencies of college students with psychological disorders and their information interaction behaviors in short videos. In comparison to adults, adolescents are more sensitive to their surrounding environment and are more susceptible to self-evaluation and external influences [55].From the interview results, we can find that for psychological disorders college students, their own pressure are academic pressure, social pressure, and worries about future work. When college students fail to reach the expected academic goal, they will have a negative cognition of their own ability, and they are easy to adopt negative attribution methods to immerse themselves in short videos to temporarily get rid of the school pressure [45]. Research indicates a positive correlation between negative life events and depression, anxiety, rumination, and suicidal ideation [56–58]. College students said that they were timid, lonely, and even afraid of communicating with their classmates, teachers, and others due to the negative events in their life. Short video interactions met the daily communication needs of college students with psychological disorders, because communicating in a virtual environment can make them feel more relaxed and more pleasant than in a real life. Most college students with psychological disorders said that college courses were obscure and they were tired of learning. Watching short videos could bring them spiritual pleasure. Therefore, they consider short videos as a crucial method to alleviate the psychological stress caused by negative life events and mitigate their adverse effects [59, 60].

During their college years, individuals often encounter various sources of stress related to interpersonal interactions, family, and the social environment, resulting in negative emotions [61, 62]. College students said that their negative emotions mainly came from interpersonal relationships, study pressure, and parents’ expectations. Studies have shown that college students have the higher expectations on their academic performance, the more stress, anxiety, and depression they will get. These factors had a negative impact on their academic performance and personal happiness [63]. In the current information age, the use of internet applications is considered a way to alleviate stress [64–66]. Short videos align with the psychological needs of college students with psychological disorders, providing relief from the pressures of a fast-paced life, and mitigating negative emotions [54]. This, in turn, promotes information interaction. College students with psychological disorders find it challenging to alleviate their negative emotions in real life. Engaging with short videos allows them to express themselves and connect with others. When they receive comments, likes, and encouragement from others, it provides them with a sense of psychological pleasure and achievement. This makes short videos an essential means to cope with life stress and regulate negative effects.

Social support can be divided into material and spiritual aspects, which are closely related to the individual’s psychological condition [67]. The classic buffering hypothesis of social support suggests that social support can reduce the negative effects of stress on mental health [68]. People with low social support were 5–6 times more likely to develop anxiety symptoms than those with high social support [69]. Social support is recognized as one of the critical social factors in predicting individual depression [70]. Research has demonstrated that the perception of social support can accurately predict depression levels in university students. Those who perceive less social support are inclined to experience higher levels of depression [71, 72]. Users can perceive others and their own existence when they are interacting with others in the virtual space [21], to obtain emotional satisfaction and psychological identification. Research has revealed that individuals use social media platforms to seek companionship, and this can directly or indirectly influence their information interaction behaviors [73, 74]. College students with psychological disorders are sensitive to external stimuli, they lack social support, care and support. Therefore, they tend to seek trust and support from others in short videos, thus to gain a sense of identity and satisfaction, resulting in a persistent short video interaction behaviour. According to the cognitive-behavioral model of pathological Internet use [75], individuals without good interpersonal communication tend to have negative beliefs. The process of information interaction usually accompanied by users’ experience and perception, which manifests as subjective psychological feelings generated by users during the process of acquiring and using information [76]. College students with psychological disorders are particularly sensitive to information, and it is easy for them to withdraw and often feel lonely and empty. Due to a lacking of effective social support, they tend to misinterpret information or make one-sided interpretations, which can cause depressive symptoms.

Post-short-video-watching behavior is one of the significant factors contributing to the continuous information interaction of college students with psychological disorders. When college students are faced with academic, life, and social pressure, watching short videos has become a way for them to relieve their stress [77]. Short video platforms provide personalized content to users according to the analysis of their preferences to stimulate continuous browsing behavior and interaction [43]. Psychologically disabled college students can establish and maintain contact with others in short videos, from which they can obtain virtual social-emotional support and eliminate loneliness [78]. Then they can promote the information interaction behavior. College students said that there were subjective preference tendencies in the process of short video information interaction, because they can choose their preferred bloggers and content based on their interests. Studies have found that interaction with short videos reduces individuals’ self-evaluation and triggers negative emotions and negative self-cognition [79]. As a result, college students who are experiencing psychological difficulties are more likely to display impulsive and extreme behaviors. Psychopathological research indicates that individuals who have exhibited symptoms of mental disorders, such as depression, anxiety, loneliness, and low self-esteem, are at a higher risk of developing addiction to the internet and smartphone applications [80–82]. College students expressed that due to a lack of effective social support in their real-life interactions, they resort to use short videos as a means to compensate for this absence. Nevertheless, excessive utilization of mobile social media platforms can result in various adverse consequences, such as anxiety and depression [83]. In the context of short videos, users frequently assume virtual personas for the purpose of exchanging information. However, the disparities and conflicts between these virtual roles and real-life identities can give rise to psychological crises, potentially leading to dissociative identity disorder or other intricate personality disorders.

Virtual social satisfaction is a crucial incentive for the short video information exchange behavior of college students with psychological disorders. Short videos offer a platform that enables users to concentrate on the content of other users and engage with them, fulfilling their emotional needs [43]. As suggested by several scholars, utilizing the internet to maintain social relationships can offer a momentary respite from stress and anxiety [84]. During the interview, it was discovered that college students with psychological disorders were primarily motivated to engage with short videos in order to attain respect and recognition from others as they expected. College students with psychological disorders expressed that engaging in activities such as leaving comments, liking, following, enchanging private messages, and sharing content provided them with an outlet to release their emotions, alliviate pressure, and fulfill their virtual social and emotional requirements. Meanwhile, the utilization of short video platforms facilitate the creation of personal social networks, enable individuals to freely articulate their personal sentiments and viewpoints. This consequently met their inherent necessity for self-expression within the virtual realm [51]. To obtain emotional resonance in the virtual environment, psychologically impaired college students shared personal experiences and provided support to people with similar backgrounds through short video interactions [85]. Short videos break the limits of time and space, connect participants to other similar individuals for solace [78], and meet the emotional acquisition needs of psychological disorder college students in the virtual environment. Research has shown that the internet has become a way to escape the stress accumulated offline [86]. To cope with stress in life, individuals may choose passive coping styles [87], such as being immersed in a short video, It satisfies the tendency of reality avoidance in the virtual environment of college students facing psychological dilemmas.

Negative cognitive tendency, negative emotions, lack of social support, post-viewing behavior of short videos, and social satisfaction in the virtual environment collectively constitute the interactive behaviors among college students with psychological disorders, which also interact with each other. Decreased interests in learning implies a loss of focus to studying, leading to academic decline and the development of negative cognitive tendencies. When teenagers fail to reach expectations, they are prone to experience academic disengagement [88], which can subsequently result in stress [89] and the emergence of negative emotions. The accumulation of excessive negative emotions can strain interpersonal relationships of college students with psychological disorders, leading to a lack of social support and poor social connections. Insufficient interpersonal communication skills can contribute to feelings of loneliness and depression among college students, prompting them to seek fulfillment of their social needs through the consumption of short videos. Chen et al. [90]have found a correlation between psychological distress and the extent of internet usage. Furthermore, when college students become engrossed in virtual environment, disengaging from the real world can lead to a stark disparity between their authentic selves and their online personas, subsequently fostering discontentment with reality. Consequently, this may make them susceptible to emotions such as frustration, depression, and other negative sentiments, ultimately resulting in negative cognitive patterns.

This article explores the psychology and interaction processes of college students engaging in interpersonal interaction and interaction through short video platforms. Rich primary data was collected through semi-structured interviews. Subsequently, the Grounded Theory method was utilized to construct a theoretical model, which focuses on the influencing factors of college students with psychological disorders in their short video information interaction. This model not only supplements the research literature on medical information with more individualized interview data but also provides an important preliminary research foundation for future cross-sectional or longitudinal studies on information interactions among college students with psychological disorders.

## Conclusion

This study focused on a sample of 30 college students with psychological disorders and employed in-depth interviews to examine the factors that shape their behaviors in the context of short video information interactions. A theoretical model was formulated to gain a comprehensive understanding of the motivating factors driving these behaviors. The findings ofthe research revealed that during short video information interactions, students with psychological disorders encounter negative emotions, negative cognition, limited social support, and exhibit social withdrawal, avoidance, anxiety, and depression. Furthermore, the study identified a reciprocal relationship between self-negative cognitive tendencies, the absence of social support, post-short video behavior, and social gratification in virtual environments as intrinsic factors influencing the short video information interaction behaviors of these students. These significant findings present theoretical underpinning that can aid universities, relevant government agencies, and short video platforms in developing effective management strategies and interventions to prevent and address psychological crises faced by college students. However, it’s important to acknowledge the study’s limitations, as it is primarily centered on college students involved in short video information interactions. Thus, it may not provide a comprehensive representation of users from diverse ages groups and backgrounds. Expending the demographics of this study population would significantly enhance the generalizability and applicability of the study’s conclusions. In addition, it is important to acknowledge that the organization of data in this study may have be influenced by subjective biases of the researchers. Therefore, future research endeavors could consider integrating quantitative research methodologies and experimental approaches, followed by rigorous empirical analysis.

### Electronic supplementary material

Below is the link to the electronic supplementary material.

Supplementary Material 1

## Data Availability

Our data were collected for the research group and are not publicly available. The datasets used and/or analysed during the current study available from the corresponding author on reasonable request.
