# Supplementary material for: Research on the mechanism of short video information interaction behavior of college students with psychological disorders based on grounded theory
Source: BMC Public Health. 2023 Nov 16;23:2256. doi: 10.1186/s12889-023-17211-4 (PMC10652505; doi:10.1186/s12889-023-17211-4)
Supplement: Supplementary file 1 — Supplementary Material 1 [file 12889_2023_17211_MOESM1_ESM.docx]

Table A1 Open coding results

| **Initial concept** | **Source data statement** |
| --- | --- |
| Demeaning self | Although I have poor acdemic performance, I still want to work hard after watching inspirational videos. but with my weak self-control ability, I can’t insist on study and lose my intersts. |
| Fear of failure | Sometimes I really want to prove myself, but I’m afraid that I can’t do it and others will laugh at me. |
| Low self-esteem | Comparing with other students, I Feel that I’m are not pretty or fit enough, and I don’t have good academic performance |
| Life trifles | There will be some small friction between roommates and classmates. If I can't handle it well, I will be very sad and reluctant to talk to them. |
| Poor acdemic performance | In the final exam, if I see someone gets higher grades than me. I will feel sad. |
| Bad interpersonal relationship | Your roommates will be rude to me for no reason, and then I will be very angry. Sometimes they are very noisy in the dormitory. Sometimes they snore at night, I can't sleep at night and feel uncomfortable. |
| Academic pressure | Since I have no foundation in my major, I may have some grammar problems that I really don't understand, so I feel powerless. |
| Passive learning | Every time I review or study for the exam, I will feel that it is not an active learning, it is passive learning for grades. |
| Lack of interest in learning | The course is boring and difficult. It takes a lot of time to memorize. Many of the technical terms are difficult to understand and can’t arise my intersts. |
| Learning maladaptation | College is different from high school. It's hard to settle down to study because I can't adapt myself. I always want to watch videos. |
| Worry and repression | Feeling confused about the future, not knowing what to do, worrying about what to do in the future, feeling that I do not have a bright future and hopeless. |
| Frustration and depression | I don't know why I feel bored or depressed. I just don't want to talk. I want to stay alone and watch videos. |
| Anxiety and tenterhook | Another anxiety is that I feel like I'm wasting my time because I have less knowledge, which is sad. |
| Big data push | Short videos will feed you whatever you like, and if you don't like them it will feed you less. |
| Personalize Content | I have my own favorite bloggers in all kinds of fields, such as cooking, photography, travel, fashion. I like all of them and I watch all of their viseos. |
| Official release | I will believe and watch all the news that released by the official websites. |
| Content quality | If the content of the short video is original or thoughtful, I will follow the blogger. |
| Shooting groups | I care a lot about the people who shoot the video, (whether it is a group or an individual), and I prefer individual shooting |
| Document content | If the content is good, I will prefer to watch. |
| Professional knowledge | If the person shooting the professional video, it will attract me to watch the video. |
| The Author's background | The short video is particularly good, with better picture quality and clarity. Then I will think that he is a writer with more efforts. Then I may choose to trust and follow the blogger. |
| Practicability | When I see the live broadcast of my favorite bloggers, I will click it to watch, choose what I need to buy, and learn science knowledge. |
| Entertainment | I think watching short videos is mainly to relax, and sometimes I will learn something on it, such as beauty makeup, then they recommend clothes, and some knowledge, such as some first-aid knowledge. |
| Uninstall the short video software | I can't control the time I spend watching the video, and I feel that it is a waste of time, so I will uninstall the short video. |
| fear of missing out | Once I browse the short video, I can’t stop, and I just want to watch it when I have nothing to do. After uninstalling, I feel boring and anxious, and download it again, over and over again. |
| Set up youth mode | In order to control the amount of time I spend scrolling, I set a teen mode, so I can spend proper time on it. |
| Report, block, and ban | When encountering bad short videos, such as those with violent tendencies, they may directly report and block them, and then they will not watch the videos with low quality. |
| Family support | The family is so busy that they hang up on the phone every time they call. |
| Friends support | I dare not tell my classmates and family about private things, because I feel embarrassed and afraid that my privacy will be revealed. |
| Support from others | There are some things I will not tell my teachers. There is still a distance between me and the teachers, and I think they will not understand if I say it. |
| Emotional support | When I see similar short videos, I will send private messages to the blogger and ask him to give me some advice. Some people will reply to me and I will feel much better. |
| Liking | When I find something I like, I'll give them a thumbs up to encourage them. |
| Comments | In short videos, I don't have to communicate with others face to face, and I'm not afraid of saying something wrong. Anyway, they don't know who I am, and I can express my ideas. |
| Messaging blogger privately | Sometimes when I see a similar experience, I will send a private message to the blogger, telling him or her about my own experiences then encouraging each other. |
| Empathizing | Always be attracted to short videos, watching short videos will make me happy, I will be infected with happiness. |
| Understanding Other people's Experiences | Some videos will arise my sympathy if the blogger have the similar experience with me. |
| Emotional identification | Some emotion descriptions are similar to my own, I will feel that what they said is very right, we get psychological agreement. |
| Psychological recognition | Won't spend all my free time on short videos. For some questionable short videos, I will verify them according to the actual situation. Watching short videos will bring me convenience. |
| Emotional satisfaction | When I meet some legal things (real-name reported events) of the short video, I have a certain judgment, according to what the blogger said, I will search on Baidu, and then discuss with classmates or teachers. |
| Avoidance | I don't want to think too much, watching short video can make me get rid of the unhappy things temporarily. |
| Utopia | I think sometimes if I have nothing to do, I can read more books, I will do well in the exam. |

Table A2 Spindle coding results

| **Principle category** | **Initial concept** |
| --- | --- |
| Self-competence denial | Self-deprecation  Fear of failure  Inferiority |
| Negative life events | Trivia  Unsatisfactory grades  Interpersonal tension |
| Negative academic attitude | Academic pressure  Passive learning  Lack of interest in learning  Maladjustment of learning |
| Unhealthy emotions | Worry and depressed emotions  Depressed mood  Anxiety |
| Lack of social support | Family support  Friend support  Others support  Emotional support |
| Content absorption behavior | Big data push  Personalized content  Official release  Content quality  Shooting group  Copywriting content  Professional knowledge  Authors’ background  Practicality  Entertainment |
| Impulsive and extreme behavior | Uninstall the short video software  Fear of missing out  Report, block, and set teen mode |
| Self-expression | Liking  Comments  Private message blogger |
| Emotional resinance | Empathy  Understand other people's experiences  Emotional recognition |
| Emotional needs | Emotional satisfaction  Psychological identification |
| Reality avoidance | Avoidance strategy  Fantasy strategy |

Table A3 Selective coding

| **Core category** | **Main category** | **Main concept** |
| --- | --- | --- |
| Negative Cognitive Tendency | Self-competence denial  Negative life events  Negative academic attitude | Negative cognitive tendency factors affect short video information interaction of college students with psychological distress |
| Negative emotions | Negative emotions | The negative emotional factors influence the interaction of short video information among college students with psychological distress |
| Lacking of social support | Lack of social support | Social supporting factors affect short video information interaction of college students with psychological distress |
| Behaviors after watching short videos | Content absorption behavior  Impulsive and extreme behavior | Short video viewing factors affect short video information interaction of college students in psychological distress |
| Social gratification in virtual environments | Self-expression  Emotional resonance  Emotional need  Reality avoidance | Social satisfaction factors in virtual environment affect short video information interaction of college students with psychological distress |
